# Phytochemical Compositions and Antioxidant and Anti-Inflammatory Activities of Crude Extracts from *Ficus pandurata* H. (Moraceae)

**DOI:** 10.1155/2013/215036

**Published:** 2013-09-26

**Authors:** Huiqing Lv, Xiaoping Zhang, XueZhi Chen, Zhijun Xie, Changfeng Hu, Chengping Wen, Kezhi Jiang

**Affiliations:** ^1^Zhejiang Chinese Medical University, Hangzhou, Zhejiang 310053, China; ^2^College of Chemical and Material Engineering, Zhejiang University of Technology, Hangzhou 310014, China; ^3^People Hospital of Jingning, Lishui, Zhejiang 323500, China; ^4^Key Laboratory of Organosilicon Chemistry and Material Technology, Hangzhou Normal University, Hangzhou 310012, China

## Abstract

*Background*. *Ficus pandurata* H. (Moraceae) is widely used in traditional Chinese medicine as a healthy food condiment or a medicine for treatment of various diseases including inflammation. *Objective*. The purpose of the present study is to investigate the phytochemical compositions and antioxidant and anti-inflammatory activities of crude water (FPW) and ethanolic extracts (FPE) from *Ficus pandurata* H. *Methods*. Phytochemical compositions were identified by a high-performance liquid chromatography-electrospray ionization-mass spectrometry method (HPLC-ESI-MS). The antioxidant activities were evaluated by diphenylpicrylhydrazyl (DPPH) and hydroxyl radical assays, and the anti-inflammatory activities were evaluated by paw edema and levels of inflammatory mediator TNF-**α** and PGE2 in monosodium urate (MSU) crystal-induced rats. *Results*. Six compounds were identified by HPLC-MS method, and abundance of phenolics was found in FPE. The FPE showed concentration-dependent-significant scavenging of DPPH and hydroxyl radicals with IC50 values 118.4 and 192.9 **μ**g/mL, respectively. The FPE treatment significantly inhibited the paw edema and the production of TNF-**α** and PGE2 in MSU crystal-induced rats. *Conclusion*. The FPE exerted stronger antioxidant and anti-inflammatory activities which may be attributed to its high phenolic content.

## 1. Introduction 

Gouty arthritis is an intense acute inflammatory disease caused by the disorder of nucleic acid metabolism that leads to monosodium urate (MSU) microcrystals deposition in the joints [1–3]. MSU crystals depositions stimulate the generation of oxygen-derived free radicals and the release of prostaglandin E2, TNF-*α*, and interleukin-1, and so forh [4, 5]. Both humoral and cellular inflammatory mediator systems play a central role in the pathogenesis of the response to urate crystals [6]. Nonsteroidal anti-inflammatory drugs such as meloxicam and indomethacin are frequently used as first-line therapies for acute gout; however, these agents are generally effective, and they also present serious side effects such as gastrointestinal bleeding and renal toxicity. Thus, traditional herbal medicines become popular because of greater effectiveness and safety. 

Plants of genus *Ficus*, which comprises more than 800 species, are distributed in tropical and subtropical regions [7]. A number of Ficus species are used as food and for medicinal properties in Ayurvedic and traditional Chinese medicine (TCM) especially amongst people where these species grow. It is widely used to treat various diseases such as various inflammation, diabetes, tumour, and malaria [8]. *Ficus pandurata* H. (Moraceae), belonging to this genus, also called *Xiao xianggou* in Lishui District (Zhejiang, China) has the function of removing dampness and strengthening the spleen. It is also widely used to treat gout, arthritis, hyperuricemia, and indigestion in traditional Chinese medicine [9, 10]. To the best of our knowledge, little information is known about the phytochemical compositions and antioxidant and anti-inflammatory activities of *F. pandurata* H. This study aims to develop a high-performance liquid chromatography-mass spectrometry (HPLC-MS) method to analyze the phytochemical compositions of *F. pandurata* H. and assess its anti-inflammatory activities to support the claim of its traditional use. Furthermore, evaluation of its antioxidant activity is also undertaken to strongly support its anti-inflammatory activity.

## 2. Experimental Section

### 2.1. Extraction from *F. pandurata* H. 

The aerial parts of *F. pandurata* H., identified by Dr. Konglong Cheng (Zhejiang Chinese Medical University, Hangzhou, China), were collected in August 2011 from Lishui Jingning of Zhejiang Province, China. The aerial parts (1000.0 g) were extracted twice under reflux for 2 h with fifteen volumes of 65% ethanol (v/v) and water, respectively. The supernatants were concentrated to the crude extracts including ethanolic extracts (FPE) and water extracts (FPW) in a rotary evaporator and stored at 4°C until use.

### 2.2. Chemicals and Reagents

All reference compounds were purchased from the National Institute for the Control of Pharmaceutical and Biological Products (Beijing, China). The purity of the compounds was higher than 99.0%. DPPH (≥96.0%) and Vitamin C (≥99.0%) were purchased from Doulai (Japan). 

HPLC-grade formic acid was obtained from Aladdin Reagents Co., and Ltd. (Shanghai, China). HPLC-grade acetonitrile and methanol were purchased from Sigma-Aldrich (St. Louis, MO, USA) and analytical-grade ethanol and meloxicam were from Huadong Pharmaceuticals Ltd., Hangzhou, China. The deionized water was purified by a Milli-Q system (Millipore, Bedford, USA). Other reagents were of analytical grade.

### 2.3. Animals

Sprague-Dawley male rats (300 ± 20 g) were obtained from the experimental animal center of the Zhejiang Chinese Medical University (SCXK (Yu)-2005-3001, Zhejiang Province, China). They were acclimatized for a week in a standard light- and temperature-controlled room and fed with plentiful food and water. The animals were treated and cared for in accordance with the Regulations of Experimental Animal Administration issued by the State Committee of Science and Technology of People's Republic of China. The experimental protocol was approved by our departmental ethics committee.

### 2.4. HPLC-ESI-MS Analysis

A portion of FPE and FPW was concentrated and dissolved in methanol to the desired concentration of 50 *μ*g/mL dry extracts. A Thermofisher HPLC instrument (Thermofisher, USA), consisted of pumps, an autosampler, a vacuum degasser, and a diode array detector, was coupled with an LCQ ion trap mass spectrometer equipped with an electrospray ionization (ESI) source (Thermofisher, USA) under the control of Xcalibur software (version 1.4). The detection wavelength was set at 254 nm by the flow rate of 0.80 mL/min. HPLC analysis was performed with a Dionex C18 column (4.6 mm × 250 mm id, particle size 5.0 *μ*m, Dionex). The mobile phase was composed of 0.1% acetic acid aqueous solution (A) and methanol (B). The gradient elution was as follows: 0 min to 5 min, 75% A; 5 min to 40 min, 75% to 30% A; and 40 min to 45 min, 30% A. 

The HPLC eluents were analyzed by full-scan MS using the positive and negative ionization modes. The sheath gas (N_2_, 99.99%) in the ESI source was set at 25 arbitrary units; the spray voltage was set at 4.5 kV for the positive mode and −3.5 kV for the negative mode; and the heated ion transfer tube (350°C) was set at +55 V for ion introduction. In-source collision-induced dissociation (20 eV) was performed to obtain the protonated molecule or the deprotonated molecule. An ion trap pressure of approximately 2 × 10^−5^ Torr was maintained with a turbo pump. Pure helium (99.999%) was used for the trapping and collision activation of the selected ions. Each mass spectrum was the average of over 10 scans.

### 2.5. Assessment of MSU Crystal-Induced Inflammation

#### 2.5.1. Synthesis of MSU Crystals

About 4 g of uric acid was dissolved and heated in 800 mL H_2_O with NaOH (9 mL/0.5 N), adjusted to pH 8.9 at 60°C, cooled over night in a cold room, washed, and dried. Needle-like crystals were recovered and were suspended in sterile saline [11]. 

#### 2.5.2. MSU Crystal-Induced Inflammation in Animals

The dosage of standard drug meloxicam (3 mg/kg·bw) used in this study was selected. The bioenhancing dosage of crude extracts (FPE, FPW) for adults is approximately 15 g (raw material)/day; equivalently, for rats, this dosage is 3.9 g/kg·bw/d calculated by the formula that converts dosage of human into that of mouse according to the respective body surface areas.

The rats were divided into five groups and each group was comprised of six animals. Group I served as a control group. In group II, inflammation was induced by intradermal injection of 0.2 mL (4 mg) of MSU crystal suspension into the right foot pad [11]. Groups III and IV which comprised MSU-induced rats were orally administered by a gavage with extracts of FPW (3.9 g raw material/kg·bw) and FPE (3.9 g raw material/kg·bw), respectively. Group V MSU-induced rats were intraperitoneally treated with meloxicam (3 mg/kg·bw, i.p.). Extracts and meloxicam were suspended in 0.5% carboxymethylcellulose in phosphate buffered saline and administered 1 h before the MSU crystal injection which was repeated for 3 days more on a daily basis.

#### 2.5.3. Assessment of Inflammation

The inflammation was quantified by measuring the ankle thickness (in millimeter) using a digital calliper at different intervals for 3 days [12]. The differences between the thickness and volume of the paw were determined for each mouse before and 5 h, 24 h, 30 h, and 48 h after MSU injection. The results were expressed as the test value of the ankle thickness and the percentage of inhibition of the control. At the end of the experimental period (72 h), the rats were killed by cervical decapitation. 

#### 2.5.4. Effect on TNF-*α* and PGE2 Production

Blood from each SD rat was collected for serum separation. The serum was used for assaying the inflammatory mediator tumour necrosis factor-*α* (TNF-*α*) and PGE2. The levels of TNF-*α* and PGE2 were qualified by the enzyme-linked immunosorbent assay (ELISA) kits (Cayman Chemicals Company, USA) according to the manufacturer's instruction. Appropriate control experiments were performed by measuring the concentration of TNF-*α* and PGE2 without drug. In all instances, the experiments were carried out in triplicate.

### 2.6. Antioxidant Evaluation

#### 2.6.1. DPPH Radical Scavenging Activity

The scavenging activity of DPPH radical was assessed based on the method given by Gordon et al. [13]. In brief, 0.5 mL DPPH solution (0.05%, w/v in methanol) was mixed in test tubes with different concentrations of FPW and FPE (25–300 *μ*g dry extracts/mL) and incubated at room temperature for 30 min. After incubation, the absorbance was recorded at 517 nm against the blank. The DPPH radical scavenging activity (%) was calculated as follows:

(1) $$\begin{array}{c} \text{Inhibition}=\left(1-\frac{A_{\text{sample}}}{A_{\text{control}}}\right)\times 100\%, \end{array}$$

where *A*_sample_ was the absorbance in the presence of extracts and *A*_control_ was the absorbance of control. IC50 value (concentration of extract required to scavenge 50% of radicals) was determined by a graph plotted between percentage inhibition and concentration. 

#### 2.6.2. Hydroxyl Radical Scavenging Activity

The ability of the extract to scavenge hydroxyl radicals was assessed by the method of Jin et al. [14]. In brief, 1, 10-phenanthroline (200 *μ*L, 3.75 mM), FeSO_4_ (200 *μ*L, 3.75 mM), and H_2_O_2_ (400 *μ*L, 0.5%, v/v) were mixed in test tubes containing different concentrations of FPW and FPE (25–300 *μ*g dry extracts/mL). The reaction mixture was incubated at 37°C for 60 min and measured at 532 nm. The hydroxyl radical scavenging activity was determined by using (1), and IC50 values were determined. 

### 2.7. Statistical Analysis

The results were expressed as means ± standard deviation and then analyzed by Graphpad Prism 5. One-way ANOVA was used for determining the statistically significant differences between the values of various experimental groups.

## 3. Results

### 3.1. HPLC-ESI-MS Analysis

**Figure 1:**
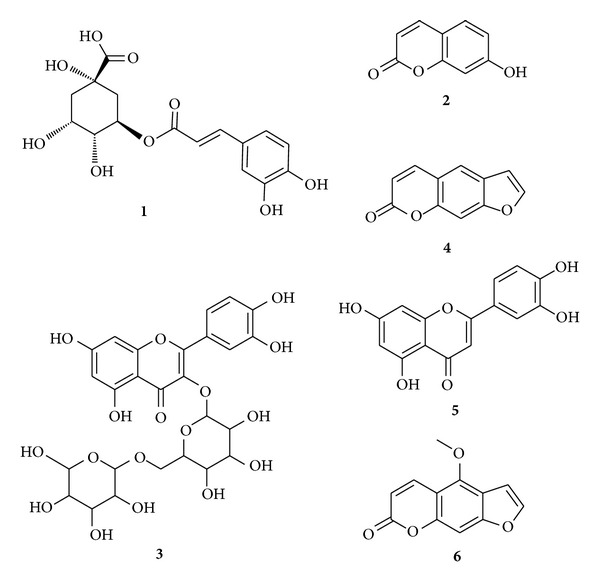
Chemical structures of the investigated compounds in *Ficus pandurata* H. plant.

According to the conditions mentioned earlier, six compounds from crude extracts were identified by ESI-MS analysis in the positive and negative scan modes (Figure 1). The ESI-MS results are summarized in Table 1. The ionization of compounds 4 and 6 were displayed better sensitivity in the positive ESI mode while that of compounds 1, 2, 3, and 5 were better in the negative mode. In-source collision-induced dissociation was performed to obtain the protonated molecule [M + H]^+^ or the deprotonated molecule [M − H]^−^ in ESI mode. 

**Figure 2:**
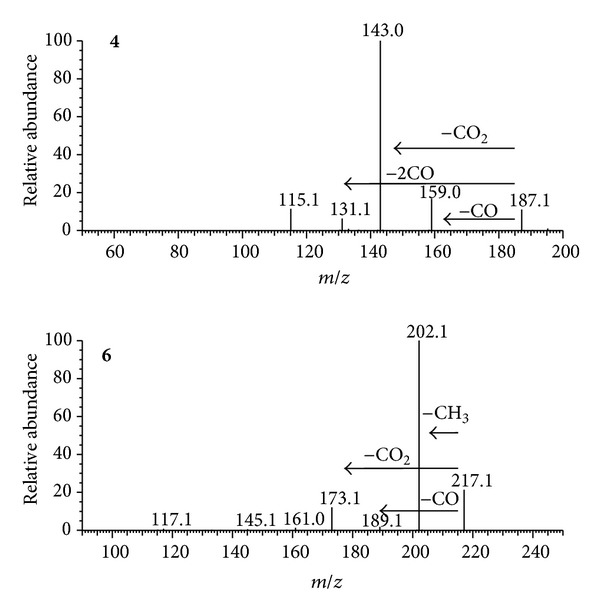
Positive MS^2^ spectra and fragmentation pathways for compounds **4** and **6**.

The positive ESI of compound **4** yielded a protonated molecule at *m/z* 187. Dissociation of the [M + H]^+^ of compound **4** produced fragments ions at *m/z* 159 (loss of CO), 143 (loss of CO_2_), 131 (losses of 2 CO), and 115 (Figure 2). Similarly, the [M + H]^+^ of compound **6** (*m/z* 217) produced fragment ions at *m/z* 202, 173, and 189, corresponding to the losses of methyl, CO_2_, and CO, respectively (Figure 2). Thus, compounds **4** and **6** were tentatively identified as psoralen and bergapten, respectively. All the six compounds including compounds **1**, **2**, **3,** and **5** (see Figure S1, Supplementary Material available online at http://dx.doi.org/10.1155/2013/215036) were applied to the HPLC-ESI-MS^2^ analysis (Table 1) for their structure identification. Identification of the six compounds were further supported by the comparisons of the UV spectra, standard substances (Figure S2), and the literatures [15]. 

Under the analytical conditions, quantitative analysis was carried out to compare the contents of the six compounds in two samples of FPW and FPE. As shown in Table 2, FPE was found to contain significantly higher amount of phenolics than that of FPW.

### 3.2. Effect of Crude Extracts on MSU-Induced Paw Edema

**Figure 3:**
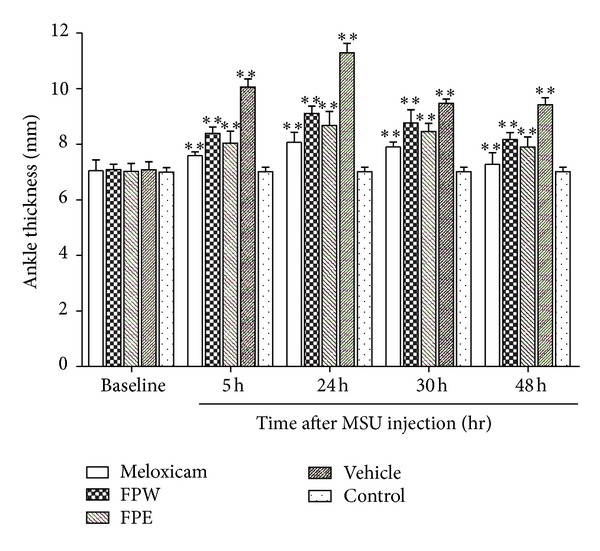
Effects of extracts (FPE, FPW, 3.90 g raw material/kg·bw) and meloxicam (3 mg/kg·bw) on MSU-induced paw edema in rats. Values are expressed as mean ± SD of six animals (*n* = 6). Comparisons were made as follows: vehicle versus meloxicam, FPE, and FPW **P < 0.01, statistical significance.

The crude extracts (FPW, FPE) and meloxicam were tested for their anti-inflammatory activity using the ankle thickness of MSU-induced rats as shown in Figure 3. The development of an edema was sustained from 1 to 48 h after MSU injection, and ankle thickness increased from 7.08 ± 0.29 mm at baseline to 11.29 ± 0.34 mm (24 h) and 9.42 ± 0.26 48 h after MSU injection, whereas meloxicam (3 mg/kg) treatment decreases the ankle diameter significantly in MSU crystal-induced rats. The FPE and FPW both at 3.90 g/kg (Figure 3) were also able to inhibit the paw edema and present significant inhibitory activity (*P* < 0.001) at 5 h with 20.1 and 16.6% of inhibition, respectively, after MSU injection. FPE has shown a stronger significant anti-inflammatory activity than FPW in this study, which may be attributed to its high phenolic content based on the chemical composition of the extracts. Our study demonstrated that *Ficus pandurata* H. was able to prevent the edema formation produced by an injection of MSU crystals in rats which confirms its traditional use.

### 3.3. Effects on TNF-*α* and PGE2 Production

**Figure 4:**
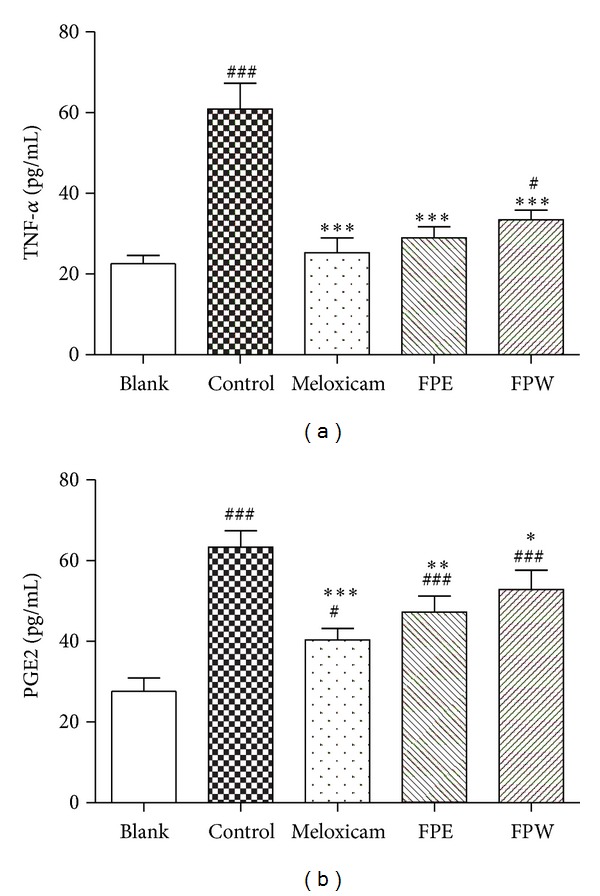
Effects of extracts (FPE, FPW, 3.90 g raw material/kg·bw) and meloxicam (3 mg/kg·bw) on TNF-*α* (a) and PGE2 (b) production in MSU crystal-induced rats. Values are expressed as mean ± SD of six animals (*n* = 6). The asterisks denote significance levels. *P < 0.05, **P < 0.01, and ***P < 0.001 in comparison to control and ^#^ *P* < 0.05, and ^###^ *P* < 0.001 in comparison to blank, and one-way analysis of variance followed by Student-Newman-Keuls' test.

As shown in Figure 4, obvious elevation in serum levels of TNF-*α* (*P* < 0.001) and PGE2 (*P* < 0.001) was observed simultaneously in the MSU-induced rats. Meloxicam at 3 mg/kg succeeded in reducing serum TNF-*α* (*P* < 0.001) and PGE2 (*P* < 0.001) levels significantly in comparison to control. FPW lowered serum TNF-*α* (*P* < 0.001) and PGE2 (*P* < 0.05) levels, while FPE reduced TNF-*α* (*P* < 0.001) and PGE2 levels (*P* < 0.01) in this model (Figure 4). Moreover, the inhibitory effect of FPE on the production of TNF-*α* and PGE2 was higher than that of FPW. These observations suggested that MSU-induced inflammation was associated with the production or expression of TNF-*α* and PGE2 levels. 

### 3.4. Assessment of In Vitro Antioxidant Activity

**Figure 5:**
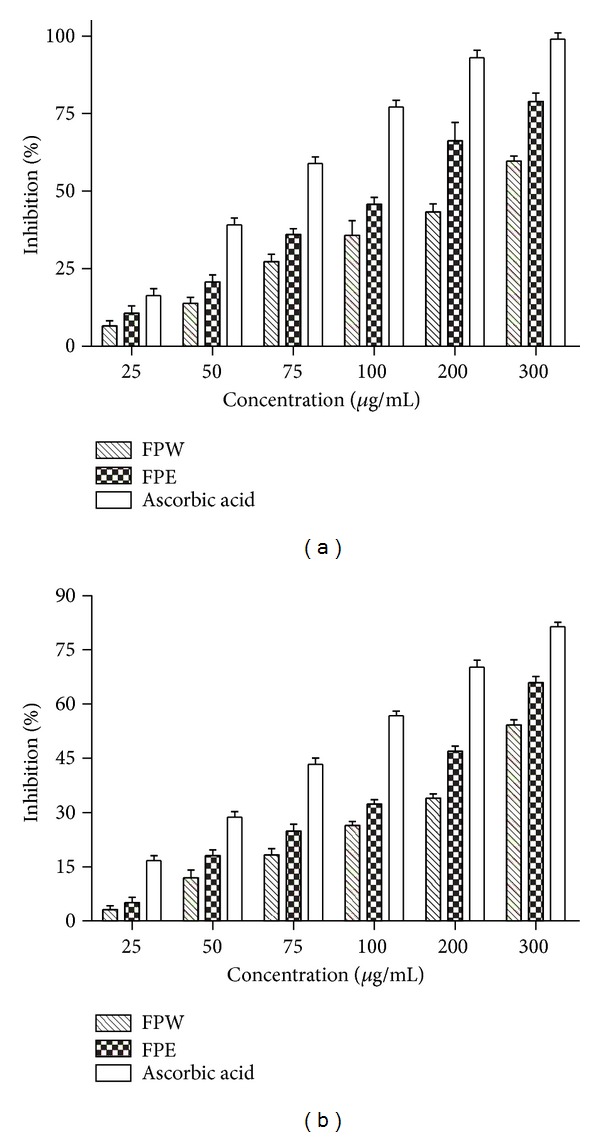
Effect of extracts (FPE, FPW, Ascorbic acid, 25–300 *μ*g dry extracts/mL) on scavenging activities of DPPH radical (a) and hydroxyl radical (b).

Multiple methods were recommended for measuring the antioxidant evaluation. The scavenging effects of DPPH and hydroxyl radicals are reliable methods for evaluating the antioxidant activities using a spectrophotometer [16, 17]. Variation in the contents of phenolic compounds, particularly flavonoids [18] and coumarins [19, 20], can affect antioxidant activities. In the present study, the scavenging activities were determined by the percentage inhibition of the formation of DPPH and hydroxyl radicals.  Figure 5(a) demonstrates significant (*P* < 0.05) DPPH radical scavenging activity of FPW and FPE; the IC50 values were found 216.9 and 118.4 *μ*g/mL for FPW and FPE, respectively. The DPPH radical scavenging activity of FPE seems to be better than that of FPW. Similarly, higher hydroxyl radical scavenging activity was found in the FPE than in the FPW (Figure 5(b)), and IC50 values for the FPE and the FPW were 192.9 and 288.2 *μ*g/mL, respectively. The scavenging abilities of DPPH and hydroxyl radical for the FPE are lower than for those of ascorbic acid, but it may be enough to remove the radical (the inhibition percentages of DPPH and hydroxyl radical were 78.9% and 65.9% at a concentration 300 *μ*g/mL). Therefore, it is suggested that the DPPH and hydroxyl radical scavenging activities in *F. pandurata* H. extracts can be attributed to their abundant phenolics, which were in agreement with those in the literatures [21].

## 4. Discussion

Gouty arthritis is caused by deposition of MSU crystals in joints. One of the most sensitive and dramatic indicators of gout is neutrophil accumulation in both the joint fluid and the synovial membrane, where a small fraction of these cells actively phagocytose MSU crystals and subsequently release mediators, reactive oxygen species, and PGE2 that are chemotactic and amplify the inflammatory reaction [22, 23]. TNF-*α* is a 17 kdD inducible peptide produced mainly by the activated macrophages and has been implicated in acute and chronic arthritic diseases [24]. Experimentally, we have reproduced it by injecting a known amount of MSU crystals in rats joints and analyzed the paw edema and the levels of TNF-*α* and PGE2. The crude extracts of FPE and FPW showed significant anti-inflammatory activity in comparison with control group when evaluated using MSU-induced paw edema in rats. In the present study, FPE has shown a stronger significant anti-inflammatory activity than FPW. TNF-*α* level in the MSU crystal-induced rats was systemically overproduced in the serum. However, the elevated levels of TNF-*α* were found to be decreased in FPW and FPE extracts as well as meloxicam treated MSU crystal-induced rats. Similarly, MSU crystal injection resulted in a significant promotion of PGE2 production. FPW and FPE extracts as well as meloxicam treatment dramatically inhibited the production of PGE2. The results suggested that the anti-inflammatory effect of FPE and FPW is probably mediated by inhibiting the release of TNF-*α* and PGE2 associated with acute gout attack. 

The pharmacological activity of a plant extract is largely dependent upon its phytochemical compositions. Therefore, we identified the compositions of FPE and FPW with a HPLC-ESI-MS method and standardized the contents of some biologically important constituents. The preliminary phytochemical analysis of crude extracts showed the presence of coumarins, flavones, and phenolic acids. Chlorogenic acid, rutin, luteolin, and/or coumarins in the medicinal plants have been known to exhibit anti-inflammatory and antioxidant properties [25, 26]. In the present study, FPE was found to contain significantly higher amount of phenolics than that of FPW. Moreover, FPE had shown a stronger significant anti-inflammatory activity than FPW, which can be related with its high phenolic content and the combined effects of its abundant phenolics. In general, anti-inflammatory and antioxidant effects of the extract may be due to the presence of chlorogenic acid, rutin, luteolin, and/or coumarins either singly or in combination.

It is well known that NASIDs such as meloxicam are effective and generally used as first-line agents to suppress acute inflammation in gouty arthritis. The results of the present study show that the inhibitory effects of meloxicam are higher than those of FPE and FPW (Figures 3 and 4). However, these agents present side effects and restriction in patients with renal or hepatic insufficiency and bleeding disorders [27]. Attributed to abundance of phenolic compounds, FPE and FPW possess both antioxidant and anti-inflammatory activities demonstrating their potential to treat a range of chronic inflammatory conditions, including arthritis. Increasing interest is focused on the potential of natural derived compounds. The specific mechanisms of action of many of these compounds remain unclear. We will further identify the real active compounds from *F. pandurata* H. extracts.

## 5. Conclusion 

In conclusion, the ethanolic extract of *F. pandurata* H. exhibited stronger antioxidant and anti-inflammatory activities, attributed to its higher phenolics content, than its water extracts that confirm the traditional use of this plant for the treatment of gouty arthritis. Further studies will be focused on the separation and purifying of active compounds and mechanisms of the pharmacological effects in *Ficus pandurata* H. plant.

## Supplementary Material

Figure S1 shows that the MS^2^ spectra and fragmentation pathways of deprotonated chlorogenic acid, 7-hydroxycoumarin, rutin, and luteolin. Figure S2 shows that the MS spectra and chemical structures of chlorogenic acid, 7-hydroxycoumarin, rutin, psoralen, luteolin and bergapaten.

## Figures and Tables

**Table 1:** HPLC-ESI/MS results of the six compounds.

Number	Retention time (min)	UV absorption (nm)	ESI-MS	Fragment ions	Molecular formula	Constituents
**1**	13.27	240, 325	353 [M − H]^−^	191 (100)	C_16_H_18_O_9_	Chlorogenic acid
**2**	22.29	325	161 [M − H]^−^	133 (100), 117, 123	C_9_H_6_O_3_	7-Hydroxycoumarin
**3**	29.00	255, 355	609 [M − H]^−^	301 (100), 300, 343, 271	C_27_H_30_O_16_	Rutin
**4**	35.11	245, 295	187 [M + H]^+^	159, 143 (100), 131, 115	C_11_H_6_O_3_	Psoralen
**5**	39.42	255, 350	285 [M − H]^−^	241 (100), 243, 217, 199, 175, 257	C_15_H_10_O_6_	Luteolin
**6**	40.66	265, 315	217 [M + H]^+^	202 (100), 173, 189	C_12_H_8_O_4_	Bergapten

**Table 2:** Contents of the six constituents.

Samples	Contents (mg/g dry extracts)					
	Chlorogenic acid	7-Hydroxycoumarin	Rutin	Psoralen	Luteolin	Bergapten
FPE	148	10	30	69	38	25
FPW	72	4	12	45	23	20

## Abbreviations

FPW: Crude water extracts from *Ficus pandurata* H.
FPE: Crude ethanolic extracts from *Ficus pandurata* H.
MSU: Monosodium urate
HPLC-ESI-MS: High-performance liquid chromatography-electrospray of flight-mass spectrometry method
DPPH: Diphenylpicrylhydrazyl.
