# Supplementary material for: Phytochemical Compositions and Antioxidant and Anti-Inflammatory Activities of Crude Extracts from Ficus pandurata H. (Moraceae)
Source: Evid Based Complement Alternat Med. 2013 Sep 26;2013:215036. doi: 10.1155/2013/215036 (PMC3804050; doi:10.1155/2013/215036)
Supplement: Supplementary file 1 — Figure S1 shows that the MS2 spectra and fragmentation pathways of deprotonated chlorogenic acid, 7-hydroxycoumarin, rutin, and luteolin. Figure S2 shows that the MS spectra and chemical structures of chlorogenic acid, 7-hydroxycoumarin, rutin, psoralen, luteolin and bergapaten. [file 215036.f1.doc]

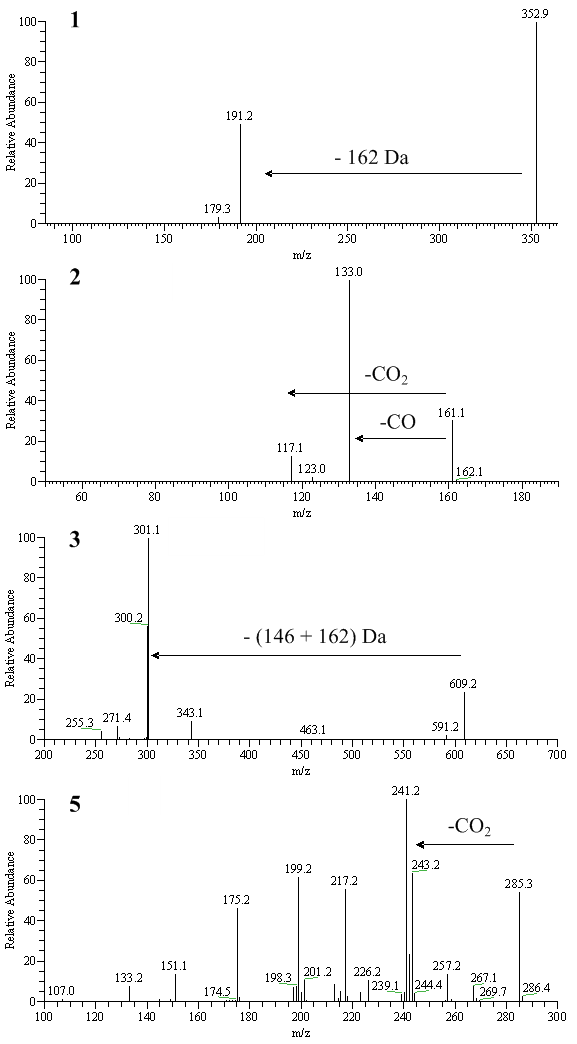


SFig. 1 MS^2^ spectra and fragmentation pathways for compound 1, 2, 3 and 5


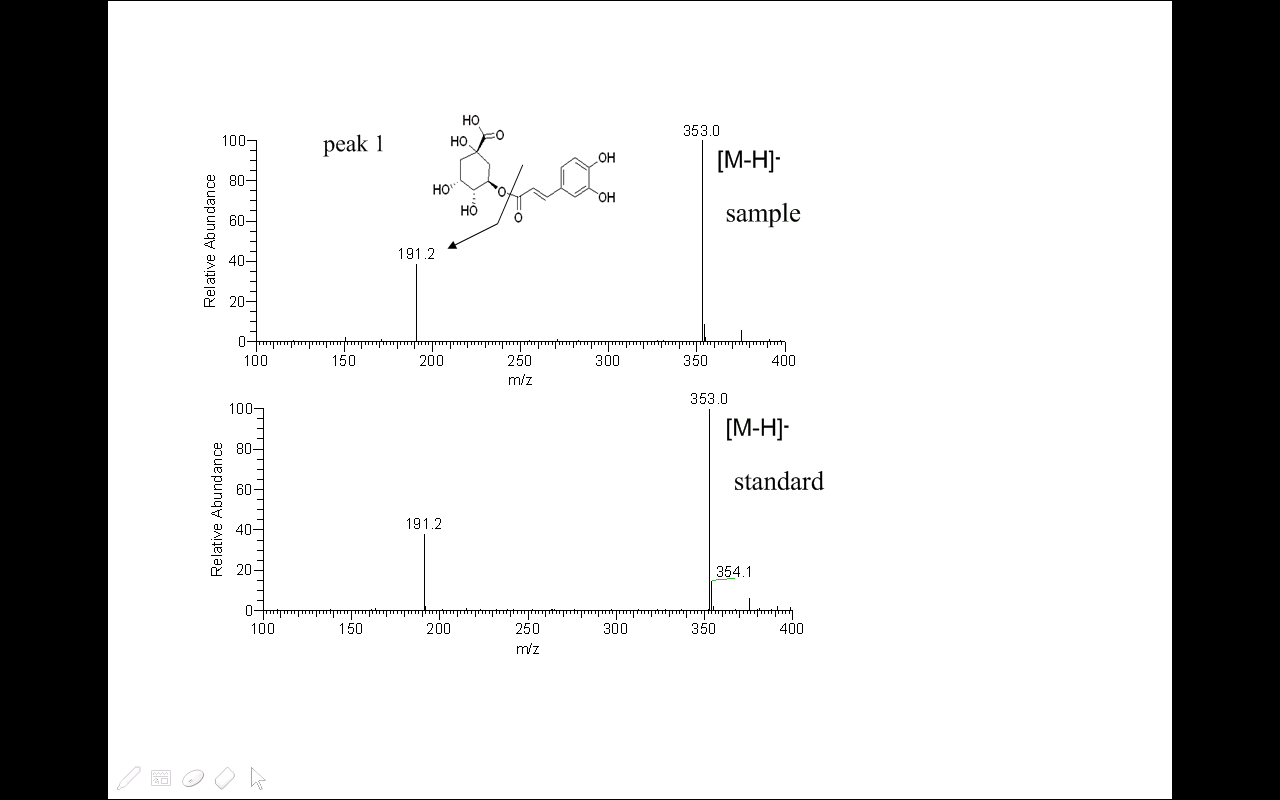

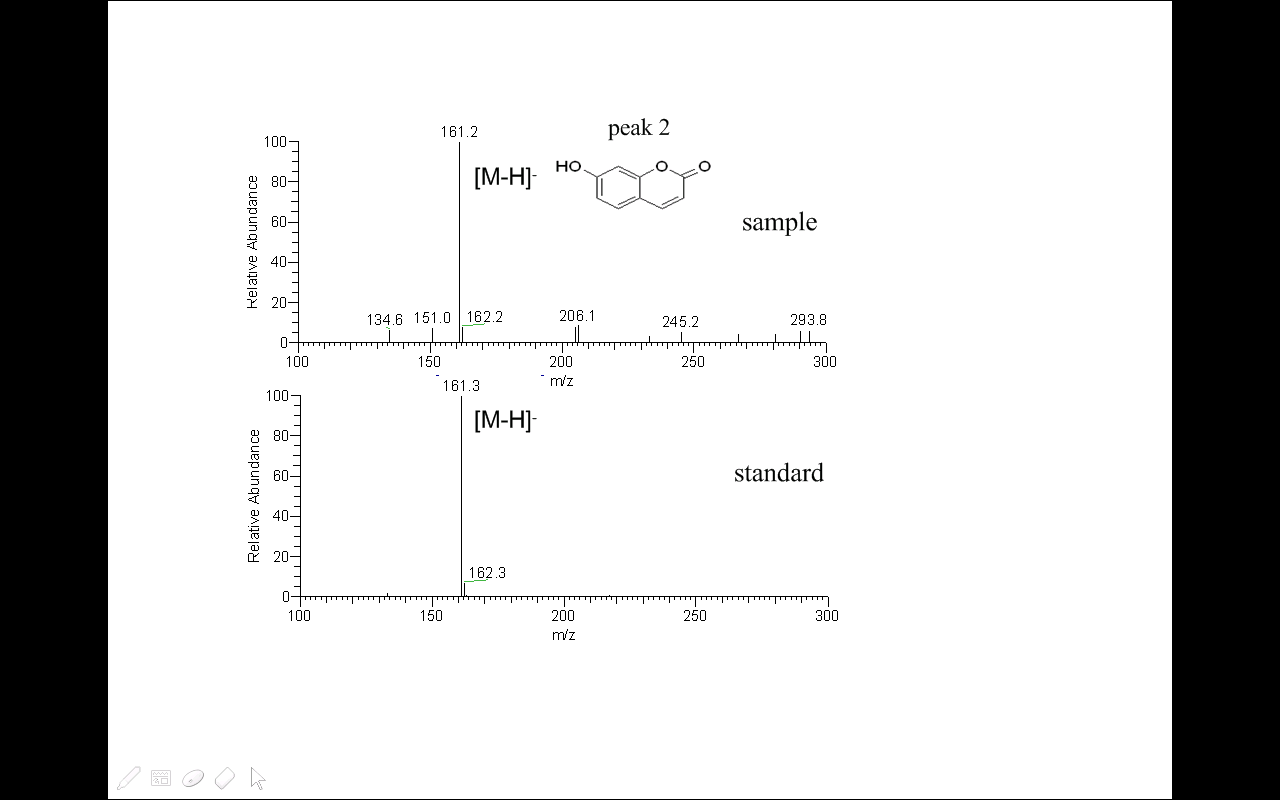


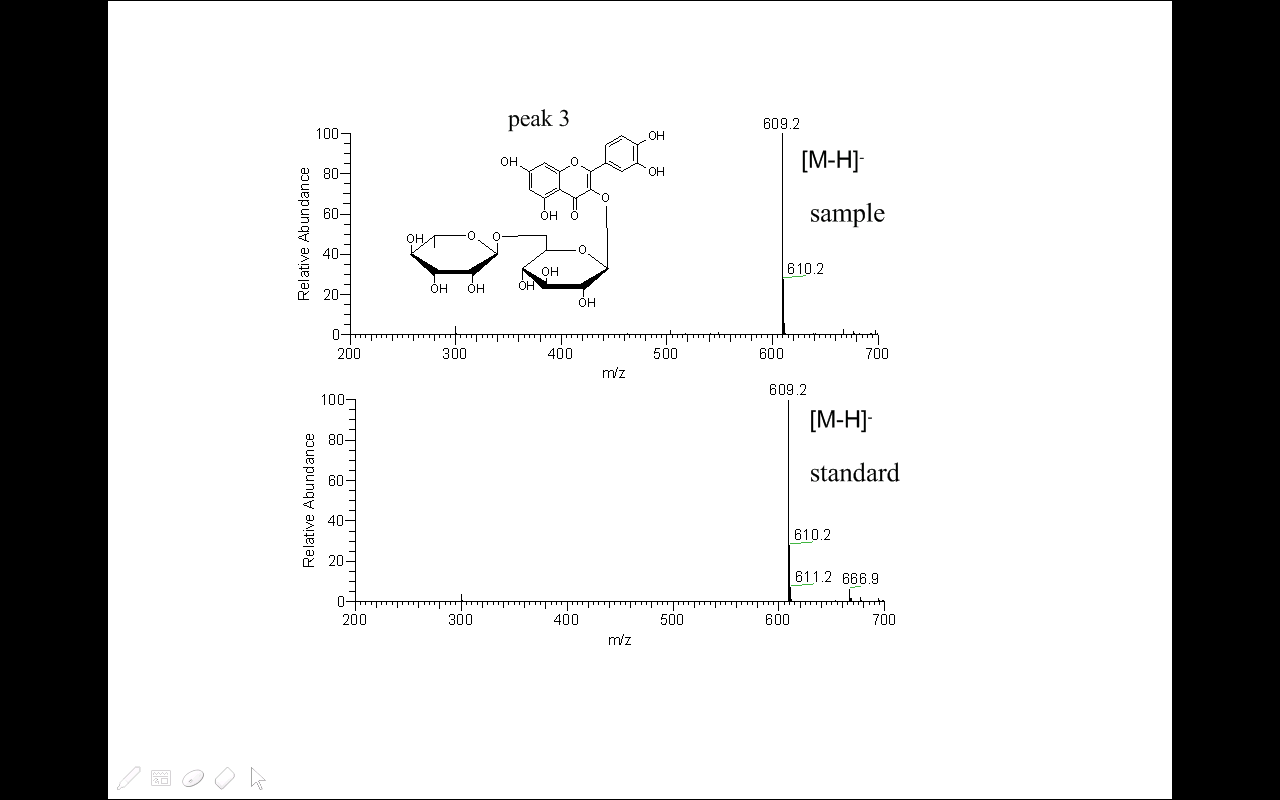

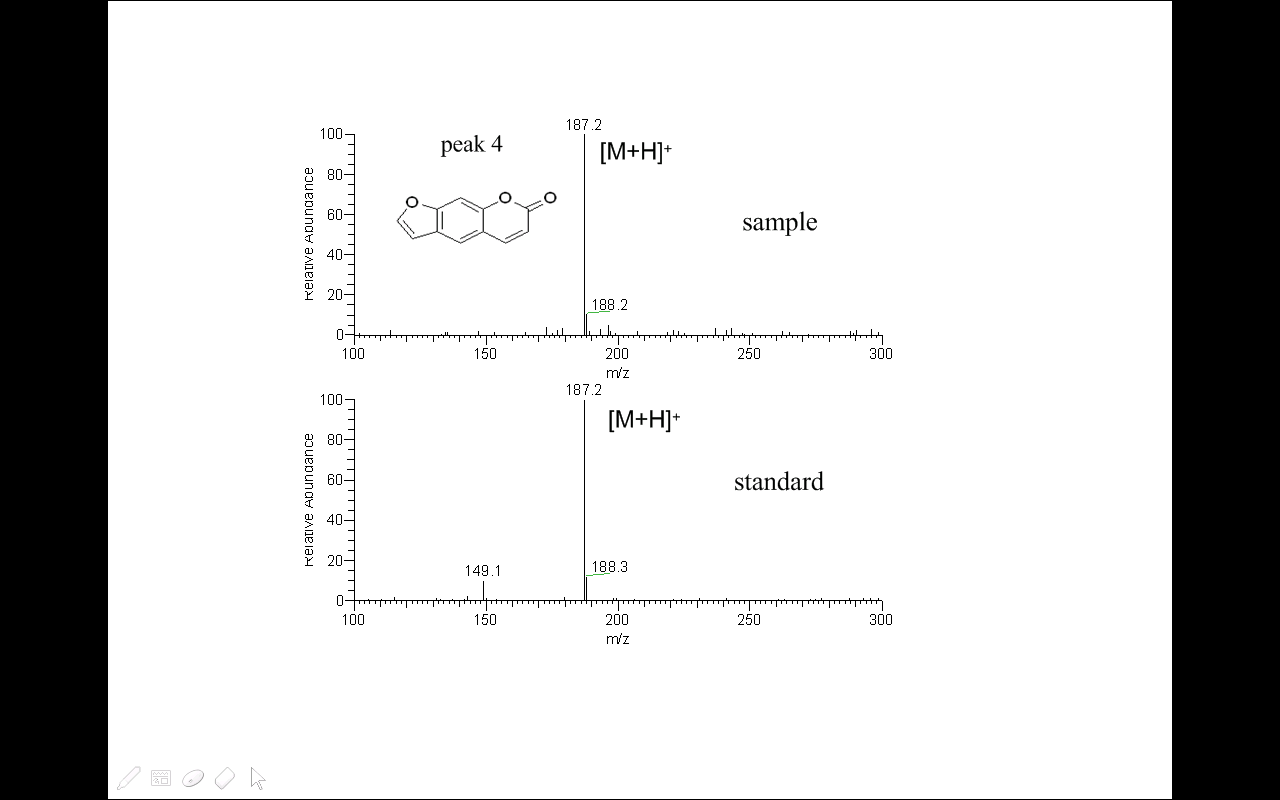


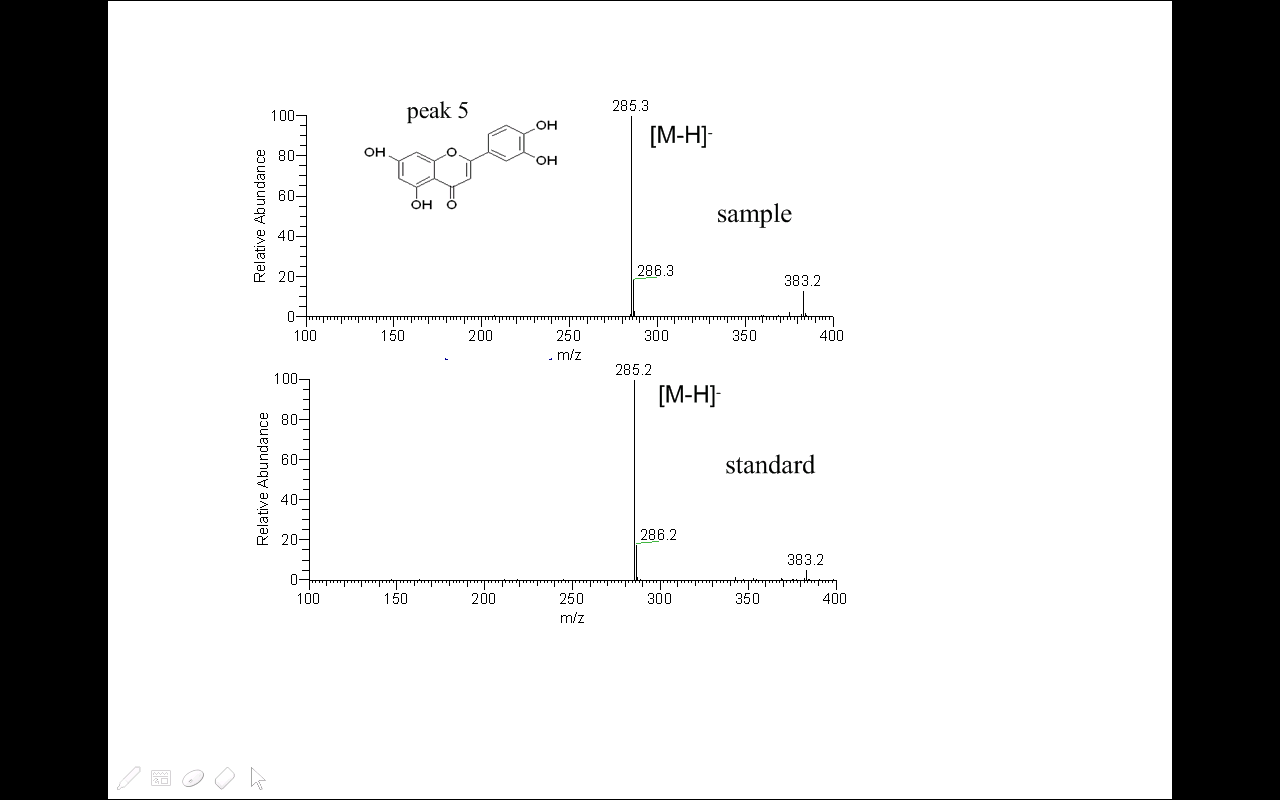

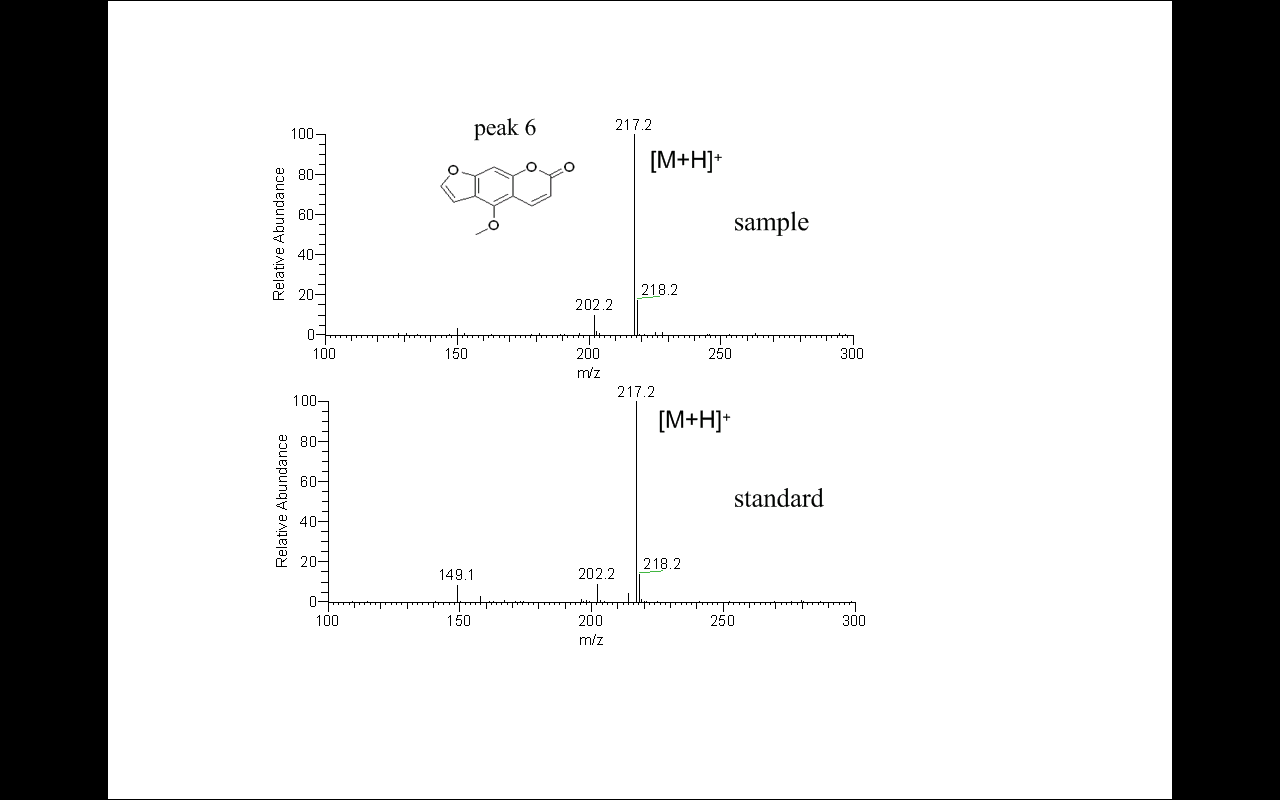


SFig. 2 MS spectra and chemical structures of peak 1, chlorogenic acid;

peak 2, 7-hydroxycoumarin; peak 3, rutin; peak 4, psoralen; peak 5, luteolin; and peak 6, bergapaten
